# Expression Patterns of Necroptosis-Related Genes: Predicting Prognosis and Immunotherapeutic Effects in Cutaneous Melanoma

**DOI:** 10.1155/2022/5722599

**Published:** 2022-07-14

**Authors:** Dong Dong, Wei Wang, Heng Wang, Liang Chen, Tianyi Liu

**Affiliations:** ^1^Department of Plastic and Aesthetic Surgery, Huadong Hospital, Fudan University, Shanghai, China; ^2^Shanghai Medical College of Fudan University, Shanghai, China

## Abstract

**Background:**

Increasing evidence has shown a strong correlation between necroptosis and antitumor immunity. However, precise expression patterns of necroptosis-related genes in cutaneous melanoma (CM) have not been clearly elucidated nor have their effects on the immune cell infiltration in the tumor microenvironment.

**Method:**

We investigated the expression patterns of necroptosis-related genes of individuals with cutaneous melanoma based on 67 necroptotic genes and methodically associated the expression patterns with the comprehensive characterization of tumor immune microenvironment. Using principal component analysis methods, the NRG score was developed to quantify the expression patterns of necroptotic genes in CM patients.

**Result:**

Three different necroptotic subtypes were determined with marked survival differences, showing distinct characteristics of immune cell infiltration. The high NRG score group with comprehensive immunosuppression was characterized by the worse immunotherapeutic efficacy and the poor prognosis, while the low NRG score group indicated a robust activation of immune function and a better response to immunotherapy, which may be responsible for a better prognosis. Furthermore, the predictive ability of the NRG score on prognosis and immunotherapeutic benefits was further revalidated using the other independent datasets of cutaneous melanoma. The results indicated that patients with low NRG scores exhibited prolonged survival. Surprisingly, all patients with CM with clinical response, including complete response/partial response, belonged to the low NRG score group.

**Conclusion:**

Our present work revealed the close association between expression patterns of necroptosis-associated genes and tumor immune microenvironment. NRG score can serve as a potential predictor to independently assess patients' prognosis with CM and effectively estimate the response to immunological therapy, thus facilitating the identification of appropriate candidates with CM for immunotherapy and the formulation of individualized therapeutic approaches.

## 1. Introduction

Cutaneous melanoma (CM) is the most fatal form of skin cancer, with metastasis at an early stage and a poor prognosis [1]. Epidemiological evidence has revealed that the morbidity of CM has increased drastically by 170% to 289,950 cases worldwide from 1990 to 2019, contributing to 80% of deaths from dermatologic cancers [2, 3]. Furthermore, CM causes approximately 55,500 deaths annually, and less than 20% of individuals with advanced CM survive more than 5 years after diagnosis [4, 5]. Over the past decade, the therapeutic landscape for advanced CM has evolved dramatically with the development of immunological therapy represented by immunological checkpoint blockade (ICB), which could effectively facilitate the reconstruction of the immune system and induce sustained antitumor immune responses [6]. However, an apparent restriction of ICB, as observed, is merely a small percentage of CM individuals with durable responses that could benefit from it, while there is no objective response for 60–70% of CM patients to immunotherapy, and 20–30% of these patients without objective response relapse with tumor recurrence and progression [7–9]. Therefore, reliable indicators or predictors are in great demand to help identify the appropriate CM individuals for immunotherapy.

Necroptosis is a new type of programmed cell death with morphological characteristics similar to necrosis relying on caspase‐independent mechanisms for the activation of death receptors, which increases membrane permeability by interacting with various phospholipids, thereby prompting the releases of chemokines and cytokines and inducing inflammation and immune response [10, 11]. In the past five years, increasing shreds of evidence have demonstrated a strong association between antitumor immunity and necroptosis. Tumor cells that undergo necroptosis have been shown to be characterized by the immune system activation, particularly the antigen presentation and activation of CD8+ T cell in tumor microenvironment (TME) [12, 13]. Furthermore, numerous studies have observed the possible combinatorial effects between immune checkpoint blockade (ICB) and the induction of necroptosis in TME on promoting long-term antitumor immunity [10, 14, 15]. With a targeted immunostimulatory mechanism, a necroptotic tumor cell mimicry nanovaccine has proven to increase antitumor immunity, inducing the expansion of natural killer (NK) cells and CD8+ T cell, and multiepitope T cell responses [16]. Furthermore, the antitumor effects of vaccination could be optimized in combination with immune checkpoint inhibitors (ICIs) in vivo [16]. These results indicate that the biological process of necroptosis is strongly associated with antitumor immunity, suggesting that necroptosis could be a potential immunotherapy target and the expression patterns of necroptosis-related genes might serve as an effective predictive factor of the response to immunotherapy of CM patients and the prognosis.

The present work evaluated the associations between expression patterns of necroptosis-related genes with the levels of immune cell infiltration in TME by combining genomic and transcriptomic data from TCGA and GEO-derived CM samples. In addition, three distinct expression patterns of necroptosis-related genes have been identified through unsupervised clustering, showing obvious differences in prognosis and the landscape of tumor immune microenvironment. Furthermore, in this study, a reliable scoring system, NRG score, has been constructed to evaluate expression patterns of necroptosis-related genes among individual tumors and to comprehensively assess the response to immunotherapy of CM patients, thereby assisting in the formulation of individualized therapeutic strategies.

## 2. Methods and Materials

### 2.1. CM Dataset Acquisition and Preprocessing

**Figure 1:**
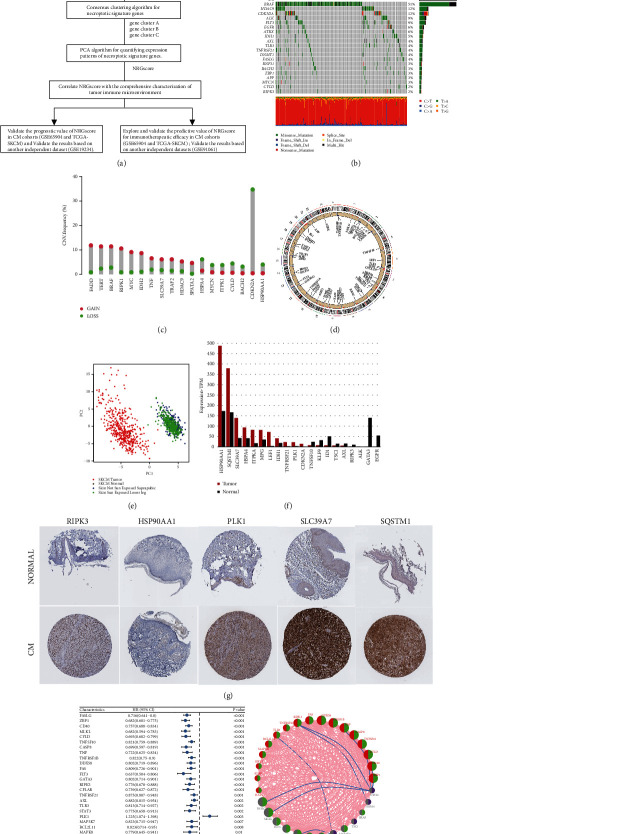
Expression and genetic variation landscapes of necroptotic genes within CM. (a) The workflow of this study. (b) Mutation frequencies of necroptotic genes (the top 20) among 467 CM cases in the TCGA-SKCM cohort. (c) Frequency of CNVs in necroptosis-related genes derived from the TCGA cohort. Column heights indicate the frequencies of variation. The green and red dots suggest deletion and amplification frequencies, severally. (d) Locations of CNVs in necroptotic genes on 23 chromosomes from patients in the TCGA cohort. (e) PCA of 67 necroptotic gene expression profiles for distinguishing tumor tissues from healthy skin and tumor-adjacent tissue in the TCGA-SKCM cohort. (f) The expression levels of necroptotic genes in melanoma versus healthy tissue samples. Differentially expressed necroptotic genes were shown (*P* value less than 0.05). (g) Immunohistochemical staining of some necroptosis-related genes (RIPK3, HSPA4, SLC39A7, and SQSTM1) in normal and CM tissues available from the HPA database. (h) In the combined CM datasets, the univariate Cox regression analyses were used to determine the importance of necroptotic genes in prognostic prediction. Horizontal length represents 95% CI of each necroptotic gene. The necroptotic gene with statistical significance (*P* value less than 0.05) was displayed. (i) Association among necroptosis-related genes in CM. The sizes of circles mean effects on the prognostic prediction of each regulatory factor.

The detailed workflow for this study is shown in Figure 1(a). First, we searched and downloaded gene expression dataset from public databases, as well as complete clinical annotation from the Cancer Genome Atlas (TCGA) database and Gene Expression Omnibus (GEO) database. Individuals that have complete survival information were selected for further analysis. Overall, 685 CM sample datasets (TCGA-SKCM and GSE65904) were identified for further evaluation. Furthermore, the independent CM datasets (GSE19234), including 44 CM individuals, were analyzed to validate the prognostic value of NRG score. In addition, an independent CM dataset (GSE91061), including 49 CM individuals receiving immunotherapy, was analyzed to identify again the predictive ability of NRG score to immunotherapy. Regarding the datasets from TCGA, RNA sequencing data, which were relative to gene expression (FPKM values), have been acquired from the University of California Santa Cruz Xena browser (Genomic Data Commons (GDC)). For the GEO datasets, we directly derived the matrix files after normalization. More specifically, we converted the FPKM values to transcripts per kilobase million (TPM) values. We corrected the batch effect from nonbiological technical bias with “ComBat” algorithm. In addition, from the TCGA database, the somatic mutation data were obtained. R Bioconductor and R (version 4.1.1) packages were used to perform data analysis.

### 2.2. Mutation-Related Genes and Mutation-Related Signatures of Tumor

A gene set was identified including 67 genes related to necroptosis through the gene set enrichment analysis (GSEA) (https://www.gsea-msigdb.org/gsea/index.jsp) and previous studies on necroptosis [17]. This study adopted the MutSigCV algorithm to determine obviously mutated genes. To be specific, MutSigCV was applied to determine distinct enrichments of non-silent somatic mutations for the single gene by removing the background mutation rate in a specific mutational context. Additionally, we used the waterfall function of the R package “maftools” to describe the mutational landscape of SMG and genes related to necroptosis in the TCGA-SKCM cohort. The CNV landscape of 67 necroptosis-related genes present in 23 pairs of chromosomes was described with the “RCircos” package.

### 2.3. Clinical Validation by Immunohistochemical Staining

The expression patterns of some representative necroptotic genes (RIPK3, HSP90AA1, PLK1, SLC39A7, and SQSTM1) in normal and CM tissues were clinically validated using immunohistochemical staining derived from Human Protein Atlas (HPA) (https://www.proteinatlas.org/) [18]. These immunohistochemical images of this study were obtained from CM patients aged 53 to 88. The antibodies used for these images are as follows: RIPK3 (HPA055087, Sigma-Aldrich), HSP90AA1 (CAB002058, Sigma-Aldrich), PLK1 (HPA053229, Sigma-Aldrich), SLC39A7 (HPA053999, Sigma-Aldrich), and SQSTM1 (CAB004587, Sigma-Aldrich).

### 2.4. Unsupervised Clustering for Necroptosis-Related Genes

The prognostic values of these genes related to necroptosis in patients with CM were revealed by the univariate Cox regression model. We used the unsupervised clustering analysis to determine different subtypes of necroptosis and divide CM individuals for subsequent analysis, based on 67 genes related to necroptosis. The stabilities and number of clusters were identified via a consensus clustering algorithm. Previous processes were carried out via the ConsensusClusterPlus package [19], and the stability of categorization was guaranteed via 1000 repetitions.

### 2.5. Differentially Expressed Genes (DEGs) in Distinct Necroptotic Subtypes

Based on selected genes related to necroptosis, individuals with CM were divided into three distinct necroptosis clusters. The empirical Bayesian approach was utilized to determine DEGs among distinct necroptosis clusters. In addition, the significance filtering criterion to identify DEGs was determined as the adjusted *P* value less than 0.001.

### 2.6. Estimation of the Infiltration of Immune Cells of TME via TIMER Database, Single-Sample S-Gene Set Enrichment Analysis (ssGSEA), and Deconvolution Algorithms

We made use of the “CIBERSORT” package to quantify the levels of infiltrating of various immune cells in CM for 1000 permutations. Furthermore, stromal/immune cells (stromal/immune scores) were evaluated using the ESTIMATE algorithm [20]. The infiltrating levels of various immune cells within TME were also assessed by the single-sample gene set enrichment analysis (ssGSEA) algorithm. The gene panels applied to label diverse immune cell types of TME were acquired through the study by Charoentong et al. [21]. The relative abundances of various immune cell types in the TME were denoted via the enrichment score determined by the ssGSEA. In addition, the TIMER database (https://cistrome.shinyapps.io/timer/) provides detailed information for systematic investigation of immune infiltration in various types of tumors [22]. Based on the gene expression profiles, a previously described algorithm was used in the TIMER database for estimating the levels of immune cell infiltration [23]. We applied TIMER to evaluate the relationships of RIPK3 with the infiltrating levels of several subtypes of CD8+ T cells.

### 2.7. Gene Set Variation Analysis (GSVA) and Functional Annotation

The distinctions in biological processes among three immune clusters were further evaluated by GSVA, which was an unsupervised and nonparametric approach to assess the variations in activity of biological process and the signal pathways in the samples [24]. An adjusted *P* value less than 0.05 was deemed as significant statistically. Using the clusterProfiler R package, the functional annotation of gene ontology (GO) and the Kyoto Encyclopedia of Genes and Genomes (KEGG) analysis of DEGs were also performed, with a cutoff value corresponding to false discovery rates <0.05. We downloaded gene sets from the MSigDB database for analyzing the correlations of RIPK3 with immune-related pathways.

### 2.8. Generation of NRG score

DEGs determined from distinct necroptosis clusters were first normalized across all samples, and the overlapping DEGs were selected. Individuals were divided into distinct subtypes through the unsupervised clustering analyses for subsequent analysis according to the overlapping DEGs. We then used the consensus clustering algorithm to identify the quantity and stability of three gene clusters. Furthermore, the prognostic analysis for every gene of this signature was conducted with the univariate Cox regression, and we extracted the genes with the prominent prognostic value for subsequent analysis. Next, the principal component analysis (PCA) was performed to establish immune gene signatures, with principal components 1 and 2 being the signature scores. The superiority of the approach was the focus of the score of this set containing significantly well-associated or anticorrelated genes, but downweighing the contribution from genes which do not associate with others of the set. The approach of defining the NRG score in our study was similar to that performed for the GGI [25]:

(1) $$\begin{array}{c} \text{NRG score}=\sum \left(\text{PC}1i+\text{PC}2i\right), \end{array}$$

where *i* represent the levels of necroptosis-associated genes.

### 2.9. Quantification of the Immune Response Predictor: Immunophenoscore (IPS)

IPS is a validated factor developed by Charoentong et al., to predict the response to anti-CTLA-4 or anti-PD-1 therapy, as it quantifies immunogenicity determinants of tumors and characterizes the immune landscapes within the tumor and cancer anti-genome [26]. The ESTIMATE algorithm, using distinct transcriptional patterns to infer tumor purity and cellularity, was used to determine stromal/immune scores for predicting the infiltrating levels of stromal/immune cells [27]. Tumor tissues with significant immune cell infiltration meant a higher IPS and lower tumor purity.

### 2.10. Statistical Analysis

The R 4.1.1 software was applied for all statistical analyses in this study. Spearman's and distance correlation analyses were applied for obtaining the correlation coefficients in two variables. The one-way ANOVA and Kruskal–Wallis tests were utilized to assess the differences across three groups [28]. The optimal cutoff point for each group, using the survminer R package, was identified based on the association between the NRG score and the patients' survival. In addition, we utilized the surv-cutpoint function of the “survival” package to tautologically examine all cutoff points for identifying the maximum rank statistic, which helped dichotomize the NRG score, and next separated individuals with CM into the low and high NRG score groups. Using the Kaplan–Meier method, the survival curve was depicted so as for prognostic analysis, and the log-rank tests were applied to examine the significance of variations. Furthermore, independent prognosis-related factors were determined by the multivariate Cox regression. Patients who had complete clinical information were selected to perform a more comprehensive multivariate prognostic analysis. Clinicopathologic characteristics including the RIPK3 expression correlated with the overall survival of CM individuals from TCGA analyzed using the Cox regression. Additionally, the data from the multivariate prognostic analysis for NRG score in the CM cohort were visualized using the forest plot R program. All statistical *P* values were bilateral, and *P* values <0.05 were deemed significant statistically.

## 3. Result

### 3.1. Landscape of Genetic Variation of Necroptosis-Related Genes in Cutaneous Melanomas

Finally, in this study a total of 67 necroptosis-related genes were identified. We first summarized the incidences of copy number variations (CNVs) and somatic mutations of 67 necroptosis-related genes in CM. Among 467 samples, 359 exhibited mutations of necroptosis-related genes, with a frequency of 76.87%. The top twenty genes, which have the highest rate of mutations, are shown in Figure 1(b). Notably, BRAF had the highest mutation frequency, followed by HDAC9; however, no mutation was observed in 11 necroptosis-related genes in CM samples, including IDH2, MPG, STUB1, ID1, TNF, BNIP3, SLC39A7, SIRT2, DIABLO, SIRT3, and IPMK. We investigated the differences in the levels of these genes between mutant and normal samples to understand the effects of these mutations on expression levels of necroptosis-related genes. Statistically significant results are shown in Supplementary Figure 2. The investigation of the frequency of CNV alteration indicated significant CNV alterations in 18 of 67 regulators. The copy number amplification frequency of FADD, TERT, BRAF, RIPK1, MYC, IDH2, TNF, SLC39A7, TRAF2, HDAC9, and SPATA2 was higher than the deletion frequency, while HSPA4, MYCN, ITPK1, CYLD, BACH2, CDKN2A, and HSP90AA1 showed a more significant CNV frequency for deletions than amplification (Figure 1(c)). To understand the details of mutations influencing necroptotic regulators, we marked the sites of CNV alterations on chromosomes in Figure 1(d). Additionally, CM samples might be completely differentiated with normal samples according to levels of the 67 genes related to necroptosis (Figure 1(e)). The necroptosis-related genes with differential expression between tumor samples and healthy or tumor-adjacent are shown in Figure 1(f) (logFC >0.6, *P* < 0.05). Besides, immunohistochemical staining images of some representative necroptosis-related genes in normal and CM tissues are shown in Figure 1(g). Significant expression differences in these genes have been observed between CM tissues and normal tissues, which are in accordance with the outcomes of previous bioinformatic analyses. Obviously, the expressions of necroptotic regulators are not clearly related to CNV alteration. Thus, the genetic variations displayed above might not be the key factors contributing to differences in the expressions of necroptotic regulatory factors.

We used the univariate Cox regression analyses to determine the prognostic value of 67 necroptosis-related genes, and these necroptosis-related genes with prognostic values are shown in Figure 1(h). According to the results, most genes were favorable prognostic factors for CM patients, while PLK1 (HR = 1.225, *P* value = 0.003), HSPA4 (HR = 1.215, *P* value = 0.041), USP22 (HR = 1.204, *P* value = 0.045), and TSC1 (HR = 1.297, *P* value = 0.016) were associated with adverse effects on the survival of patients with CM. Furthermore, the comprehensive landscape of interactions between necroptosis-related genes with prognostic value, as well as the respective value in prognostic prediction of CM cases, was analyzed using the networks of the necroptosis-related genes (Figure 1(i)). The results revealed that positive correlations were observed among the overwhelming majority of necroptosis-related genes with prognostic value, while USP22 exhibits negative correlations with TNF, RIPK3, FASLG, MLKL, ZBP1, and CD40. Besides, the effects on necroptotic induction by promoting the expression of stimulators might be limited due to the obvious positive correlations between the stimulators and suppressors.

In summary, the above results indicated that the transcriptomic and genomic landscapes in necroptosis-associated genes between CM tissues and tumor-adjacent tissues or normal tissues were highly heterogeneous, suggesting that necroptosis-related genes might have vital effects on the occurrence and progression of CM.

### 3.2. RIPK3 Expression Significantly Related to Immune Microenvironment

**Figure 2:**
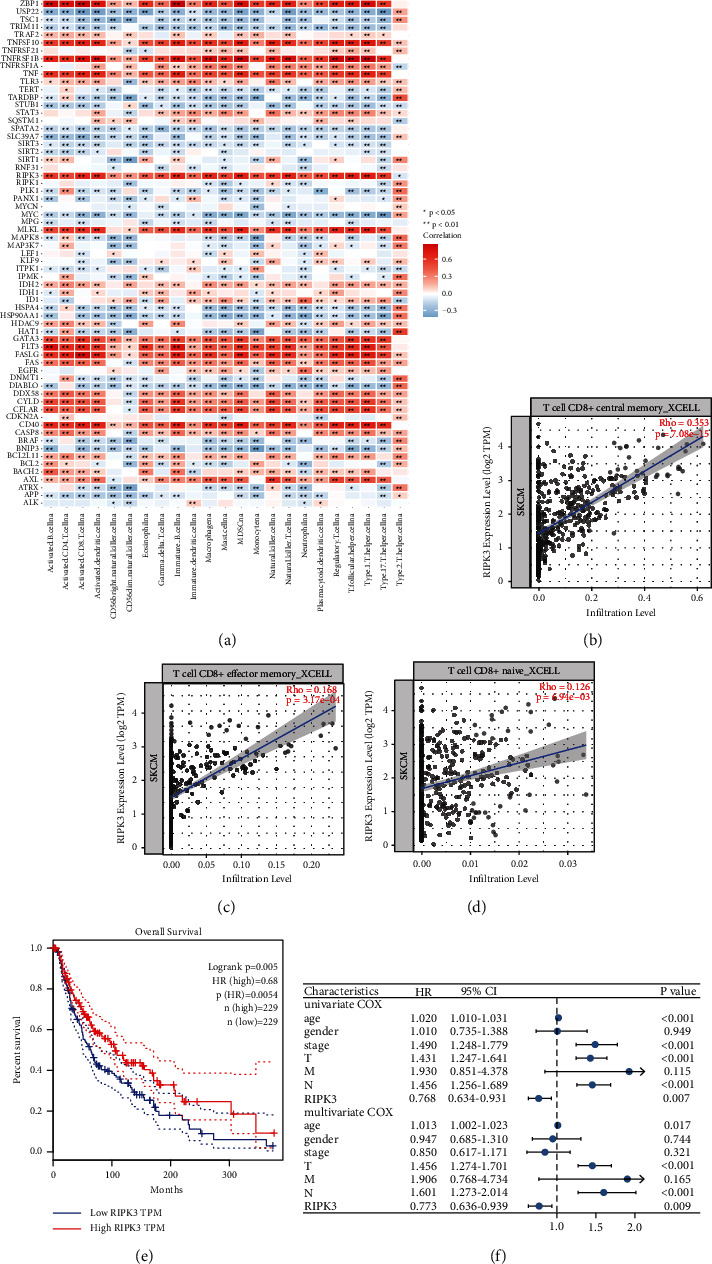
RIPK3 expression significantly related to immune microenvironment. (a) The relationships of necroptosis-related genes with the tumor-infiltrating level of different kinds of immune cells in CM tissues. (b) There are significant positive connections between RIPK3 expression and CD8+ effector memory T cell infiltration. (c) There are significant positive correlations between RIPK3 expression and CD8+ central memory T cell infiltrating levels. (d) There are significant positive relationships between RIPK3 expression and CD8+ naive T cell infiltration levels. (e) Difference in survival of CM individuals between the high RIPK3 expression group and the low RIPK3 expression group. (f) With univariate and multivariate Cox regression, correlations with survival and clinicopathologic characteristics in TCGA individuals were investigated.

Increasing evidence has revealed a strong correlation between antitumor immunity and necroptosis. Tumor cell that undergoes necroptosis has been shown to be closely linked to immune system activation. Therefore, we further explored the relationships between necroptosis-related genes with the expression patterns of infiltrating immune cells of CM tissues. The results revealed that the expression levels of necroptosis-related genes were closely associated with the infiltrating levels of immune cells in CM tissues (Figure 2(a)). Some necroptosis-related genes, such as USP22, TSC1, PLK1, and HSPA4, are significantly inversely associated with infiltrating degrees of various immune cells. These genes have been also determined as risk factors and are related to poor prognosis. Meanwhile, RIPK3 is significantly positively related to the infiltrating levels of the majority of immune cells. RIPK3 has been also identified as the favorable factors and are associated with more favorable clinical outcome (Figure 2(e)). The multivariate COX regression analysis suggested that RIPK3 is an independent prognostic factor in melanoma, along with patients' age (*P*=0.017), AJCC T stage (*P* < 0.001), and N stage (*P* < 0.001) (Figure 2(f)). Besides, we selected RIPK3, a widely used marker in necroptosis, as an example to further clarify the relationships of these immune features with necroptosis-related genes. As shown in Figures 2(b)–2(d), significant positive correlations have been observed between the expression degree of RIPK3 and levels of various CD8+ T cells, such as CD8+ naive T cell, CD8+ effector memory T cell, and CD8+ central memory T cell. The above results have been validated using various algorithms, including CIBERSORT, EPIC, XCELL, MCPCOUNTER, CIBERSORT-ABS, and QUANTISEQ (Supplementary Figure 3A–E). Prior study has shown that RIPK3 can drive the secretion of inflammatory chemokines and cytokines, thereby activating cytotoxic T lymphocyte, in the process of cell death [29]. To further detect the difference in immune infiltration related to the RIPK3 expression. The median value of RIPK3 expression was used to classify individuals into two groups (high and low RIPK3 groups). Significant differences in immune cell infiltrating levels, key immune-related pathways, expression level of antigen-presenting molecules, and immune checkpoints have been observed (Supplementary Figure 3F–H). From the above, we could speculate that RIPK3-mediated necroptosis of tumor cells may promote the activation of various tumor-killing immune cells and enhance the immune cells infiltrating within tumor tissues, thereby enhancing the intratumoral antitumor immune response. Furthermore, these results were consistent with previous studies and provided more shreds of evidence in the potential ability of RIPK3 to serve as a novel target in melanoma, especially in enhancing “hot” tumor phenotype and improving the efficacy of existing immunotherapies.

### 3.3. Identification of Necroptotic Subtypes

**Figure 3:**
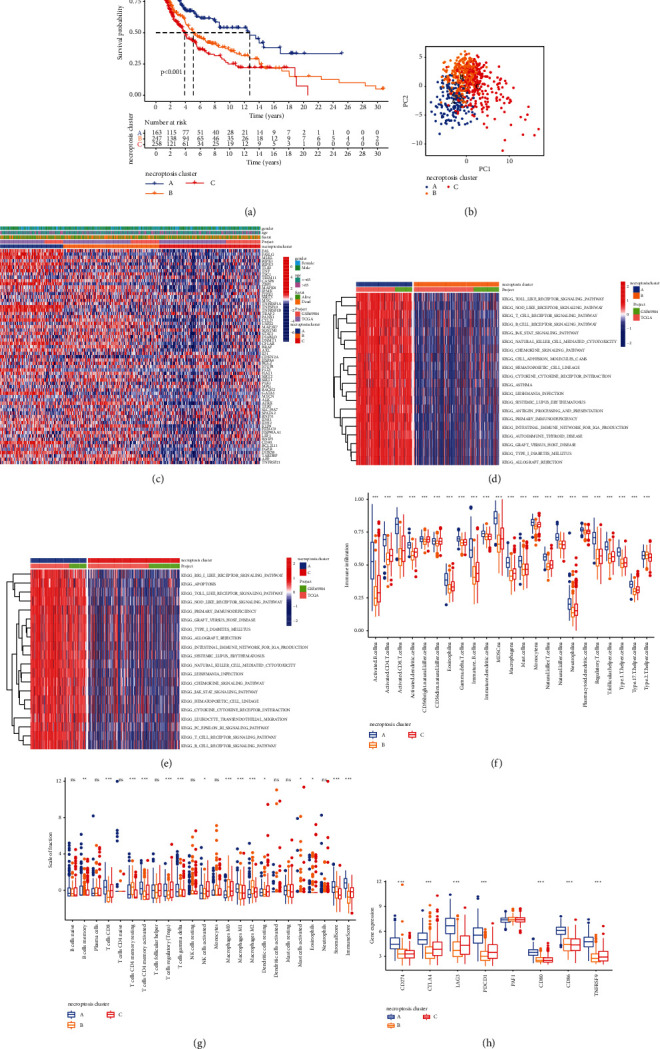
Immune landscape and functional annotation of different necroptotic subtypes. (a) Survival analysis of different necroptotic subtypes in the combined CM cohort. The K-M curves with a *P* value less than 0.001 suggested that the differences in survival were significant across the three clusters. Immune cluster A displayed superior survival compared with the other subtypes. (b) PCAs for transcriptome profile of necroptotic subtypes, suggesting an obvious distinction in the transcriptome among different subtypes. (c) Unsupervised clustering of the genes related to necroptosis in the combined CM cohort. Necroptosis cluster, age, and status of survival served as patients' annotation. Red means high levels, and blue means levels. (d), (e) GSVA enrichment analyses of the activated signaling pathways in three different necroptosis clusters. Red color means the activation of the signaling pathway, and blue means the inhibition of the signaling pathway. (f) Variations in the abundance of infiltrating immune cells among necroptosis clusters A, B, and C using “ssGSEA.” “^*∗*^” represents the statistical *P* value (^*∗*^*P* value less than 0.05; ^*∗∗*^*P* value less than 0.01; ^*∗∗∗*^*P* value less than 0.001). (g) Difference in the abundance of infiltrating immune cells among necroptosis clusters A, B, and C using “CIBERSORT” analysis. “^*∗*^” represents the statistical *P* value (^*∗*^*P* value less than 0.05; ^*∗∗*^*P* value less than 0.01; ^*∗∗∗*^*P* value less than 0.001). (h) The expression of immune checkpoint genes in three necroptosis clusters.

To further explore the potential biological features of different expression patterns of necroptosis-related genes in CM, this study divided the cases into different necroptotic subtypes based on the levels of 67 necroptosis-related genes. A total of 686 tumor samples that have available clinical data and OS information profiles based on the meta-cohort (GSE65904; The *Cancer* Genome Atlas (TCGA)-SKCM) were enrolled in the analysis. Using the ConsensusClusterPlus package of R software, unsupervised clustering analyses were conducted to divide individuals with CM into three separate subtypes with significant survival differences (Supplementary Figure 1). According to the prognostic analysis, the necroptosis cluster A showed a particularly noticeable survival advantage among three different necroptosis clusters, while the necroptosis cluster C had the worst prognosis (Figure 3(a)). Additionally, PCA demonstrated the obvious differences among three different necroptosis clusters relative to the transcriptional profile of 67 necroptosis-related genes (Figure 3(b)). According to the heat map analysis, the expression levels of 67 necroptosis-related genes of necroptosis cluster C individuals were obviously lower compared with that in patients of necroptosis cluster A and necroptosis cluster B (Figure 3(c)).

### 3.4. Immune Landscape and Functional Annotation of Different Necroptosis Clusters

To investigate the biological characteristics among three different necroptosis clusters, we performed the GSVA enrichment analysis. Compared with necroptosis clusters B and C, necroptosis cluster A presented remarkable enrichment of signaling pathways, which were associated with immune activation and apoptosis, including B cell receptor signaling pathways, T cell receptor signaling pathways, Toll-like receptor signaling pathways, chemokine signaling pathways, and cytokine-cytokine receptor interaction signaling pathways, indicating that necroptosis cluster A exerted powerful immune activity, which was consistent with the results of prognostic analysis (Figures 3(d) and 3(e)). The robust immune function of necroptosis cluster A could be a plausible explanation for discovered correlation between high expressions of necroptosis-related genes with better survival.

To further investigate the relationship between expression patterns of necroptotic genes and immune function, the components of immune cells in TME among three necroptotic subtypes were analyzed. The ssGSEA analysis, as expected, revealed that various infiltrating immune cells were prominently enriched in necroptosis cluster A, including CD4+ T cell, MDSC, macrophages, activated B cell, mast cell, gamma delta T cell, eosinophils, CD8+ T cell, and natural killer cell (Figure 3(f)), while necroptosis cluster B was distinguished by comprehensive suppression of immune function. We further assessed the proportion of different subtypes of infiltrating immune cells of CM based on the “CIBERSORT” method. The results also indicated higher levels of immune effector cells in necroptosis cluster A, including M1 macrophage and memory CD4+ T cell and activated CD8+ T cell, which were consistent with the above analyses of ssGSEA (Figure 3(g)). The composition of the TME was also assessed via reliable ESTIMATE algorithm. Furthermore, we calculated the stromal/immune scores to verify the degree of infiltration of stromal/immune cells. The results indicated that cluster A displayed the highest immune score and the highest stromal score, while cluster B displayed the lowest immune scores, and cluster C had the lowest stromal score (Figure 3(g)). Furthermore, we assessed the expressions of several vital genes associated with immune checkpoints, including CTLA-4, PD-L1, LAG3, PAF1, PD-1, CD80, CD86, and TNFRSF9 in each necroptosis clusters. In necroptosis cluster A, the expressions of these genes except PAF1 were obviously higher than that of necroptosis cluster C or cluster B (Figure 3(h)).

In summary, the expression patterns of necroptosis-related genes were strongly associated with immune function. Significant differences were observed in immune status between three distinct necroptotic subtypes. Necroptosis cluster A with higher expression of 67 necroptosis-related genes showed stronger immune function along with better survival.

### 3.5. Construction of Necroptosis-Related Gene Signatures and Identification of Necroptotic Gene Subtypes

**Figure 4:**
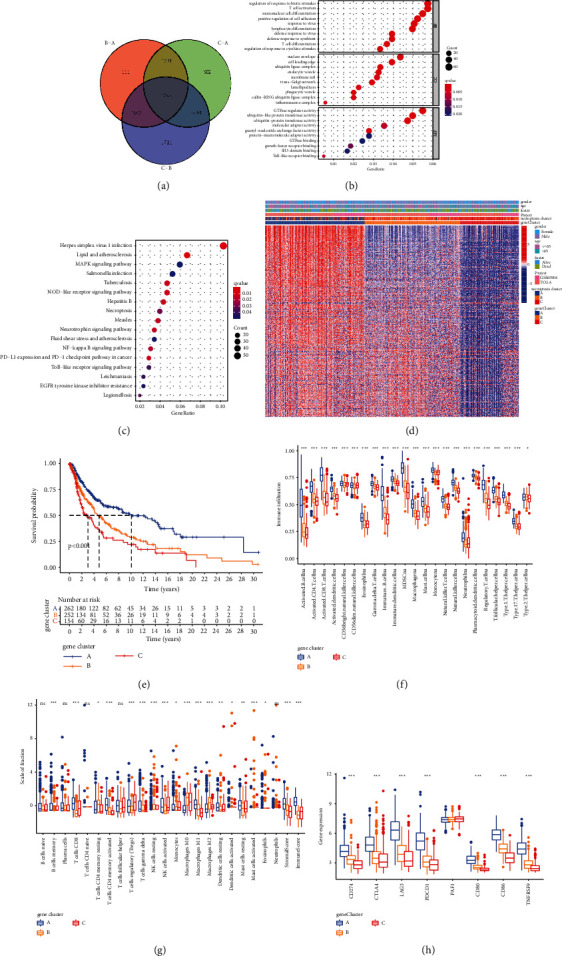
Construction of necroptosis-related gene signatures and identification of necroptotic gene subtypes. (a) Venn diagram presenting 1242 overlapping DEGs among three necroptosis clusters that were identified. (b) Functional annotations of DEGs based on GO analysis and the circle size mean the number of genes enriched. (c) Functional annotations of DEGs based on KEGG pathway analysis and the circle size represents the enriched gene number. (d) Unsupervised clustering of the necroptosis-related gene signatures in the combined CM cohort. The necroptosis cluster, gene cluster, survival status, and ages served as individual annotation. Red means high levels, and blue means levels of these genes. (e) Survival analyses of distinct gene subtypes in the combined CM cohorts. The K-M curves with *P* < 0.001 suggested that differences in survival were obvious across the three clusters. (f) Difference in abundances of immune cell infiltration among gene clusters A, B, and C using “ssGSEA.” “^*∗*^” means the statistical *P* value (^*∗*^*P* value less than 0.05; ^*∗∗*^*P* value less than 0.01; ^*∗∗*^^*∗*^*P* value less than 0.001). (h) The expression of genes related to immune checkpoints in three gene clusters. (g) Variations in abundances of immune cell infiltration across gene clusters A, B, and C using “CIBERSORT” analysis. “^*∗*^” represents the statistical *P* value (^*∗*^*P* value less than 0.05; ^*∗∗*^*P* value less than 0.01; ^*∗∗*^^*∗*^*P* value less than 0.001).

To unravel the potential biological characteristics of each necroptosis cluster, with the Limma packages, differential analyses of gene expression among three necroptosis clusters were performed to identify transcriptome distinctions, finally determining 1242 overlapping differentially expressed genes (DEGs) related to necroptosis (Figure 4(a)). Then, we utilized the clusterProfiler package to perform KEGG and GO enrichment analyses for these DEGs. As expected, these DEGs were prominently enriched in biological processes associated with necroptosis and immune function, involving lymphocyte differentiation, T cell differentiation, and T cell activation, again confirming that the expression pattern of the necroptosis-related gene played a vital role in immune modulation in TME (Figures 4(b) and 4(c)). Next, the above overlapping DEGs were utilized to conduct a survival analysis for each gene via the univariate Cox regression. A total of 527 DEGs associated with prognosis were identified (*P* < 0.05), which altogether constituted the necroptosis-related gene signatures. To better validate the above regulatory mechanism, we performed the unsupervised clustering of these necroptotic signature genes detected in three necroptosis clusters, which divided the GSE65904 and TCGA-SKCM cohorts into distinct gene subtypes (Supplementary Figure 1). Consistent with necroptotic subtypes, three distinct genomic phenotypes were recognized via an unsupervised clustering algorithm, termed gene clusters A, B, and C, severally. The transcriptome profiles of these prognostic DEGs based on gene clusters were represented as a heat map (Figure 4(d)).

The prognostic characteristics of three gene clusters were investigated by combining them with prognostic information. Overall, 266 of 300 patients with CM were aggregated in gene cluster A, suggesting better survival outcomes, whereas patients in gene cluster C (159 patients) were observed to be strongly associated with poorer outcomes. Besides, 257 patients with CM belonged to gene cluster B with an intermediate prognosis (Figure 4(e)). Furthermore, the landscapes of infiltrating immune cells in the TME have been investigated in three gene clusters using “CIBERSORT” and the “ssGSEA” methods (Figures 4(f) and 4(g)). We found that gene cluster A had dramatically higher immune scores and stromal scores compared with other gene clusters and it presented the highest activated CD8+ T cell and activated CD4+ memory T cell infiltration. As shown in Figure 4(g), gene cluster B, having much lower immune scores, was characterized by remarkable immunosuppression-related M2 macrophage infiltration. Furthermore, we also investigated expressions of some vital immune checkpoint-relevant genes in the three gene clusters to unravel the biological behaviors among different gene clusters, indicating obvious differences. Gene cluster A was related to much higher expressions of immune checkpoint genes, whereas the lower gene expression level was observed for gene clusters B and C (Figure 4(h)). In brief, the correlation between prognostic profiles and immune profiles among distinct gene clusters indicated that the sorting scheme was reasonable and scientific.

### 3.6. Establishment of the Necroptosis-Related Gene Score (NRG Score) and the Association between NRG Score and TME

**Figure 5:**
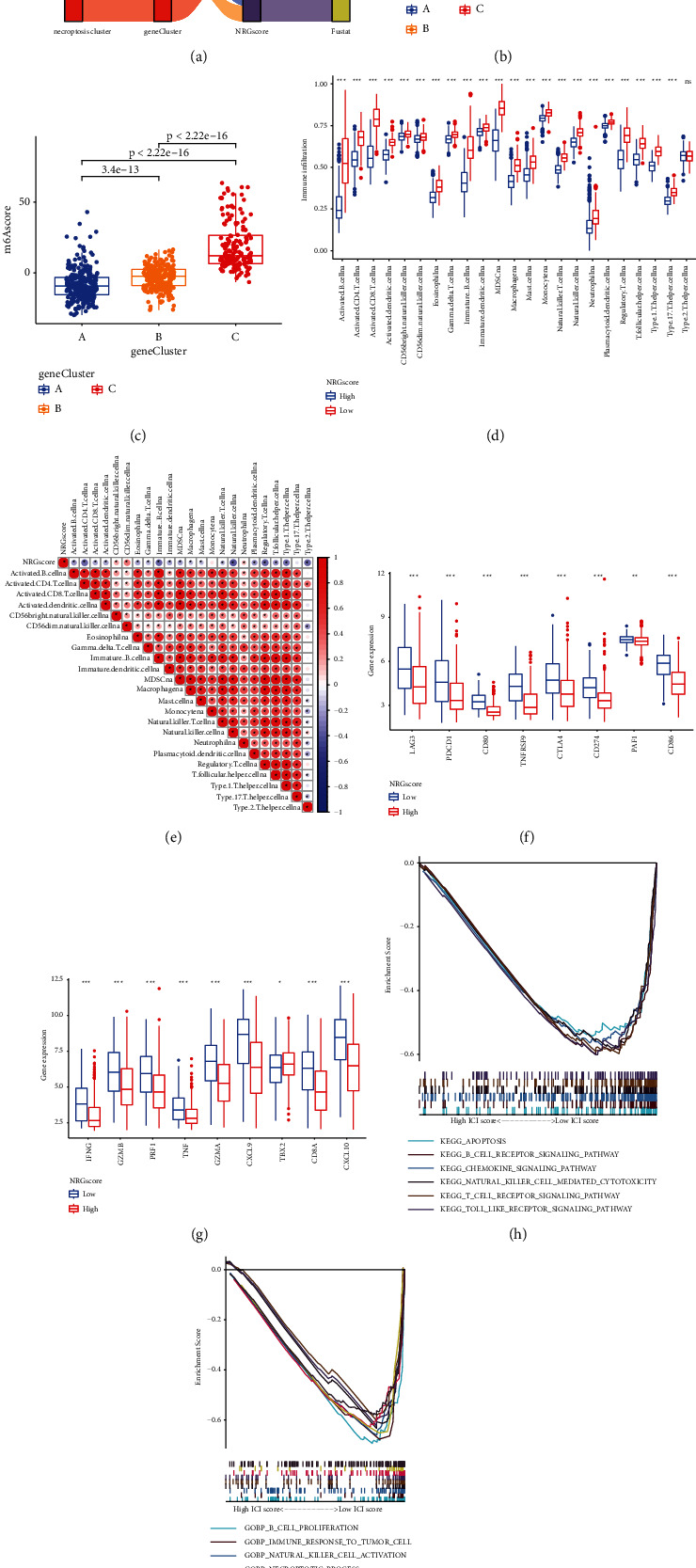
Establishment of NRG score. (a) Alluvial diagram indicating attribute alterations of different patterns. (b) NRG score differences among three necroptosis clusters (*P* value less than 0.001, Student's *t*-test). (c) Differences in NRG score across three gene clusters (*P* value less than 0.001, Student's *t*-test). (d) Differences in the abundance of infiltrating immune cells between the high NRG score and low NRG score groups using “ssGSEA.” “^*∗*^” represents the obvious *P* value (^*∗*^*P* value less than 0.05; ^*∗∗*^*P* value less than 0.01; ^*∗∗∗*^*P* value less than 0.001). (e) Association between NRG score and immune infiltrating cells of TME analyzed by Spearman's analysis. Positive and negative correlations are represented by red and blue colors, respectively. (f) The expression of immune checkpoint-related genes in the low and high NRG score groups. (g) The levels of immune activation-associated genes in the low and high NRG score groups. (h), (i) GSVAs (KEGG and GO).

The process of necroptosis is complicated and heterogeneous across different individuals. To acquire quantitative predictors of the expression patterns of necroptosis-related genes in individual patients with CM, based on the above necroptosis-related gene signatures, we developed the scoring system to quantify expression patterns of necroptosis-associated genes of individual with CM, called NRG score. Detailed constructive processes of NRG score are provided in the Methods section. To further explore the characteristics of the NRG score, we classified individuals with CM into a low or high NRG score group with an optimum cutoff value identified using survminer package. The alluvial diagram indicated the attribute alterations in different patterns. As shown, most of the patients in gene cluster B and almost all individuals belonging to gene cluster C corresponded to the high NRG score. In contrast, most patients from gene cluster A belonged to the lower NRG score group. Additionally, the necroptotic subtypes were consistent with relevant gene clusters (Figure 5(a)). The Kruskal–Wallis test further revealed remarkable differences in the NRG score among distinct gene clusters and necroptosis clusters (Figures 5(b) and 5(c)). The lowest average score was associated with gene cluster A, whereas gene cluster C was linked to the highest average score among different clusters, suggesting that NRG score might be negatively correlated with immune function. The higher score might be indicative of immune suppression, while the low score might be associated with immune activation. Next, the ssGSEA analysis demonstrated prominently higher degrees of the majorities of infiltrating immune cells in the low NRG score group, including higher infiltration levels of activated dendritic cell, MDSC, activated CD8+ T cell, activated B cell, activated CD4+ T cell, and NK cell (Figure 5(d)). Additionally, the subsequent analysis further indicated that the NRG score was significantly negatively related to the level of infiltration of kinds of immune cells, including CD4 T cell, activated B cells, and CD8 T cell, all of which further confirmed the above hypothesis (Figure 5(e)). Furthermore, we calculated the levels of several vital immune checkpoint genes, including CTLA-4, PAF1, CD80, PD-L1, LAG3, CD86, PD-1, and TNFRSF9, along with the expressions of signatures related to immune activity, such as CXCL9, TNF, PRF1, GZMB, CXCL10, IFNG, GZMA, CD8A, and TBX2. Interestingly, the Wilcoxon test revealed that most key genes related to immune checkpoints and activation of immune function were substantially upregulated in the low NRG score group, except TBX2 (Figures 5(f) and 5(g)). Furthermore, GSEA demonstrated that apoptosis signaling pathway and immune-related pathways were elevated in the low NRG score group, such as the T cell receptor and Toll-like receptor signaling pathways, NK cell-mediated cytotoxicity pathways, and B cell receptor signaling pathways (Figure 5(h)). Furthermore, among GO terms, the low NRG score group was still characterized by the activation of necroptosis-related biological processes and enrichment of immune-associated pathways such as B cell proliferation, immune responses to tumor cells, and NK cell activation, all of which further robustly indicated that the lower NRG score could mean more active immune function and stronger necroptosis than the high NRG score group (Figure 5(i)).

### 3.7. The Prognostic Ability of NRG Score

**Figure 6:**
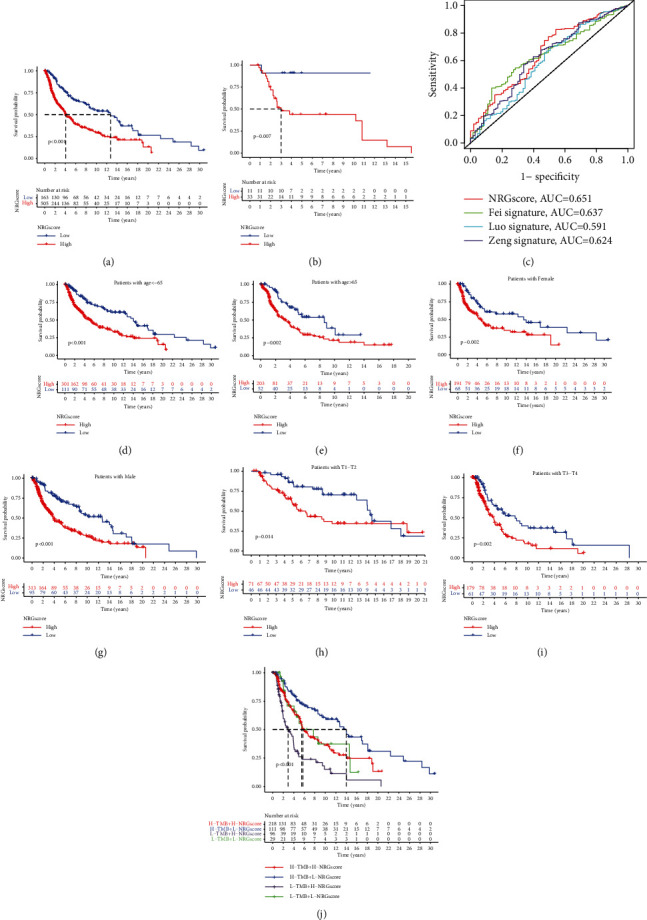
Prognostic ability of NRG score. (a) Survival analysis of melanoma patients with low and high NRG scores based on the K-M curve (*P* value less than 0.0001, log-rank test). (b) Survival analysis of melanoma individuals with high and low NRG scores from another independent dataset based on the K-M curve (*P* value less than 0.0001, log-rank test). Survival analysis of individuals with high and low NRG scores based on the K-M curves (*P* value less than 0.0001, log-rank test). (c) Comparison between the ROC curves of NRG score with that of other published models. (d) Age≤65 (*P* value less than 0.05, log-rank test). (e) Age >65 (log-rank test, *P* value less than 0.05). (f) Female individuals (log-rank test, *P* value less than 0.05). (g) Male individuals (log-rank test, *P* value less than 0.05). (h) Individuals with stages T1-T2 (log-rank test, *P* value less than 0.05). (i) Individuals with stages T3-T4 (log-rank test, *P* value less than 0.05). (j) Stratified survival analysis of patients derived from the TCGA-SKCM cohort divided according to both NRG scores and TMB (*P* value less than 0.001, log-rank test).

The subsequent analysis assessed the values of NRG score in predicting the outcome of individuals with CM. The results revealed that those with a low NRG score showed a considerable survival advantage over the high NRG score group (*P* value less than 0.001) (Figure 6(a)). Additionally, the prognostic values of NRG score were further validated based on another independent dataset of CM patients (GSE19234). As expected, the survival of patients belonging to the low-score group was also superior to the high-score group (*P* value less than 0.001) (Figure 6(b)). Besides, to further explore the accuracy of NRG score in predicting the prognosis of melanoma individuals, the ROC curve of NRG score was plotted in the TCGA cohort and was compared with some other published models. As shown in Figure 6(c), the merged ROC curve indicated that AUC score of NRG score was 0.651, which was superior to that of the other models, suggesting that NRG score had a relatively accurate prognostic ability. Additionally, our present study also explored whether NRG score was an independent predictor of CM individuals' prognosis. Based on the multivariate Cox regression model analysis, the predictive ability of NRG score was revealed to be independent of patient sex (*P* < 0.001 ), age (*P* < 0.001), or ACJJ T stage (*P* < 0.01), indicating that this score system could exert its predictive effect as an independent, reliable, and effective biomarker (Figures 6(d)–6(i)).

Many studies have demonstrated that the tumor mutation burden (TMB) could influence the outcomes of CM patients and the response to ICB [30, 31]. An increased TMB is always associated with a better immune therapeutic effect and prolonged progression-free survival [32]. Considering the prominent clinical implications of TMB, the functional relationships between the NRG scores and TMB were investigated to decipher the genetic signatures of distinct immune clusters. First, based on the set point of TMB, patients with CM were divided into separate subtypes, and we observed that patients belonging to the high TMB group indicated a better prognosis compared with individuals with low TMB, as shown in Supplementary Figure 4, which was consistent with previous studies [33]. Next, we compared TMB of individuals with the low NRG score and high NRG score groups. Nevertheless, no statistical difference was observed in TMB between low and high-scoring groups (Supplementary Figure 4). Using the stratified survival analysis, our present work further revealed that the predictions based on NRG score were not affected by the status of TMB. No matter in the low or high TMB subgroups, remarkable prognostic variations were observed between the high and low NRG score groups (Figure 6(j)). To summarize, the above results further demonstrated that the NRG score was an independent predictor, which could effectively evaluate the outcomes of patient with CM.

### 3.8. The Effects of NRG Scores on Predicting Immunotherapeutic Benefits

**Figure 7:**
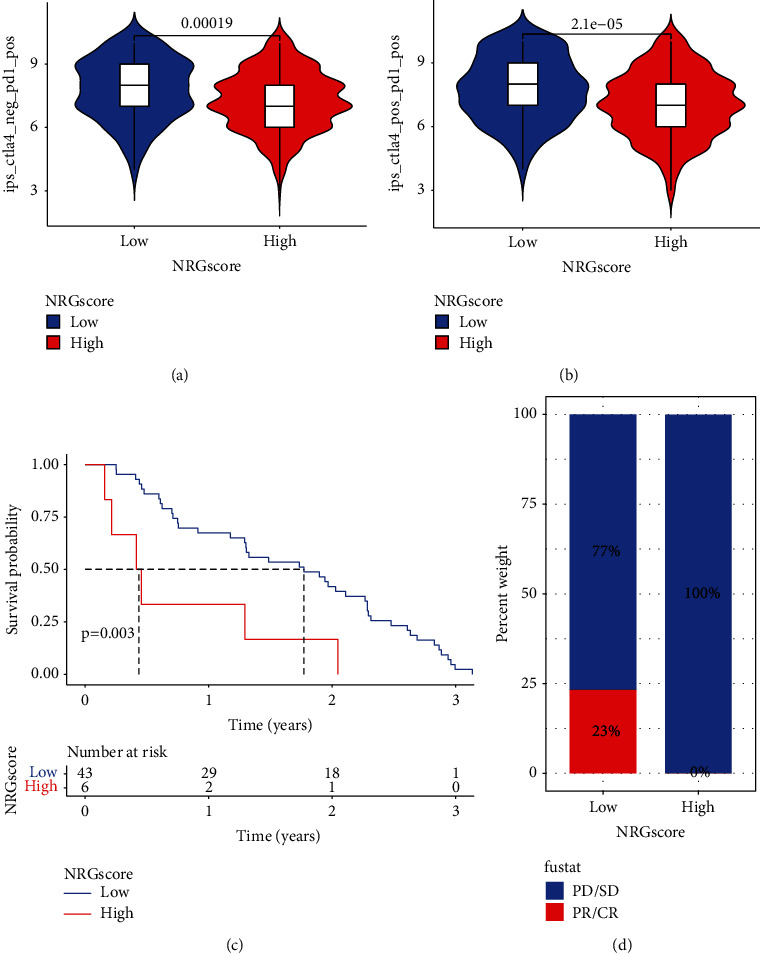
Effect of NRG scores on the predicting immunotherapeutic benefits. (a) The immunophenoscore of anti-PD-1 immune checkpoint therapies in melanoma individuals with the low or high NRG score. (b) The immunophenoscore of anti-PD-1 and CTLA-4 immune checkpoint therapy in melanoma patients with the low and high NRG scores. (c) Survival analysis of patients with low and high NRG scores from the cohort consisting of CM patients receiving immunotherapy (GSE91061) based on the K-M curves (*P* < 0.0001, log-rank test). (d) Proportions of PD-1 blockade immunotherapy-responsive patients in the high and low NRG score groups. CR, PR, and PD stand for complete response, partial response, and progressive disease.

Although ICB has achieved remarkable outcomes as cancer therapy with an unprecedented increase in patient survival, it is unfortunate that only a small percentage of CM individuals could benefit from durable responses, whereas most patients experience little clinical benefit. The effects of the NRG score in evaluating the response of CM patients to ICB were validated in the subsequent analysis. Based on the immunophenoscore developed by Charoentong et al. to predict response to immunotherapy [26], we found anti-PD-1 immunotherapy alone or the combination of anti-PD-1 and anti-CTLA-4 immunotherapy, and the immunophenoscore was always higher in the low NRG score group than in the high NRG score group in the TCGA-SKCM cohort, suggesting that patients belonging to this group could benefit from these two types of immunotherapies (Figures 7(a) and 7(b)). To further validate this speculation, we used another independent dataset consisting of CM patients receiving immunotherapy (GSE91061) to explore this predictive effect of NRG scores on immunotherapeutic benefits. The results indicated that individuals with low NRG scores exhibited a prolonged survival compared with high-scoring patients (Figure 7(c)). Surprisingly, further analysis indicated that in this immunotherapy cohort, all patients with clinical response, including partial response (PR)/complete response (CR), belonged to the low NRG score group, suggesting that NRG scores were extremely sensitive in predicting immunotherapeutic benefits (Figure 7(d)). Collectively, these findings robustly suggested that the NRG score could serve as an immunotherapeutic and prognostic biomarker, thereby assessing the immunotherapy response.

## 4. Discussion

Immunotherapy symbolized by ICB has brought revolutionary advances in the fields of cancer therapies, contributing to an unprecedented increase in patient survival [7, 34]. To date, the US Food and Drug Administration has approved ICIs targeting three different molecules (CTLA-4, PD-1, and its ligand, PD-L1) for use in humans, improving CM patients' prognosis [35, 36]. However, an obvious limitation of ICB therapy is merely a minor percentage of CM individuals that will achieve durable responses from this treatment, whereas the majority will experience little clinical benefit, which is far from meeting clinical needs [37]. Consequently, it is extremely necessary to identify appropriate individuals with CM as candidates for immunotherapy.

Increasing shreds of evidence have shown that necroptosis, one of the new forms of programmed cell death, has a vital effect on inflammation, antitumor responses, and antitumor immune responses, which involve immune system activation, including antigen presentation and CD8+ T cell cross-priming in the TME [38, 39]. In addition, the possible synergistic effects between the induction of necroptosis in TME and the ICB have been observed to promote durable antitumor immunity, further supporting the close correlation between the necroptotic process and the immune response to the tumor. Therefore, necroptosis might be a potential immunotherapy target and the expression patterns of necroptosis-related genes might serve as an effective predictor of the prognosis of patients with CM and the response to immunotherapy. However, comprehensive characterizations of the immune infiltration landscape among different expression patterns linked to the expressions of necroptosis-associated genes are not recognized generally.

In this study, based on 67 necroptosis-related genes, we identified three different necroptotic expression patterns with obvious differences in the characterization of immune cell infiltration of TME and survival of patients. Heat map analysis revealed that the expression levels of most necroptosis-related genes were obviously higher in cluster A with significant survival advantage than in cluster C with the worst prognosis. Among three distinct patterns, necroptosis cluster A was featured by immune-related pathway activation and elevated levels of immune cell infiltrating, including activated B cell, CD8 T cell, NK cell, CD4 T cell, and activated DC cell, suggesting that the level of immune cells infiltrating in TME was significantly positively correlated with individual survival. These findings were consistent with previous studies [40, 41], which could also be a plausible explanation for the discovered correlation between expressions of necroptosis-related genes with the prognosis of patients.

Subsequent analysis indicated that the differences in the mRNA transcriptome between three different expression patterns of necroptosis-related genes were closely related to the necroptotic biological process and immune-related biological pathways. In particular, 1242 DEGs overlapping among three subtypes were defined using Limma packages of *R* software, and KEGG and GO analyses showed that these genes were mainly enriched in the necroptosis-related biologic process and the NF*κ*B signaling pathway, which is one of the most important necroptotic signaling pathways [42]. In addition, enrichment of these genes in immune-related biological pathways was also observed, including activation and differentiation of T cells, lymphocyte differentiation, and PD-L1 expression and the PD-1 checkpoint signaling pathway, further suggesting the close correlation between necroptosis and antitumor immunity. Then, 527 DEGs with obvious prognostic value were finally identified by survival analyses for each gene by the univariate Cox regression model, together constituting the necroptosis-related gene signatures. Consistent with the clustering analysis based on necroptosis-related genes (necroptosis clusters A, B, and C), we discovered three genomic clusters (gene clusters A, B, and C) according to the selected necroptotic signature genes, showing significant differences in prognosis and TME characterization. Further analysis demonstrated that gene cluster A with a prominent survival advantage had the highest stromal score and immune score, as well as the high immune cell infiltration in the TME, suggesting an immunoactivated phenotype. Interestingly, the degrees of infiltration of M1 macrophages, as observed in cluster A, were significantly higher compared with other subtypes, while the degrees of infiltration of M2 macrophages were the lowest. M2 macrophages can excrete many immunosuppressive cytokines, facilitating tumor progression and metastasis, associated with a poor prognosis [43]. Additionally, as targets for immunotherapy, the expression levels of several vital immune checkpoints were also investigated among three gene clusters. Gene cluster A was related to much higher expression levels of immune checkpoints, whereas gene cluster C with the worst prognosis had the lowest level of expression. These results robustly demonstrated the importance of comprehensively evaluating the expression patterns of necroptosis-related genes, which could better understand the characterization of TME and might help estimate the response to immunotherapy and prognosis of CM patients.

It is necessary to establish the scoring system to quantify expression patterns of necroptosis-related genes in individual patients with CM, considering individual heterogeneity of the necroptotic patterns. Based on the necroptotic signature genes mentioned above, this study constructed a scoring pattern termed NRG score. The subsequent GSEA demonstrated that the apoptosis signaling pathway and immune-related pathways were elevated in the low NRG score group, such as T cell receptor signaling pathways, B cell receptor signaling pathways, and NK cell-mediated cytotoxicity pathways. Moreover, among the GO terms, a low NRG score was still characterized by the activation of necroptosis-related biological process and the enrichment of immune-related pathways such as B cell proliferation, immune response to tumor cells, and NK cell activation. Furthermore, the infiltrating degrees of various immune cells in TME of the low NRG score group were much higher compared with those of the high NRG score group, all of which further indicated that the lower NRG score could mean more active immune function and stronger necroptosis, compared with the high NRG score group.

Based on the cohort consisting of TCGA-SKCM and GSE65904, we further evaluated the value of the NRG score as a prognostic predictor. The results revealed that individuals with low NRG scores showed a prolonged survival than high score patients. In addition, the above conclusion was validated again based on another independent CM dataset (GSE19234), which robustly indicated that NRG score might serve as an effective prognostic marker of CM patients. Furthermore, we explored the predictive capacity of this scoring system for the immunotherapeutic benefits of CM patients. The Wilcoxon test revealed that all key genes associated with immune checkpoints were upregulated in the low NRG score group, such as CTLA-4, PAF1, CD80, PD-L1, LAG3, CD86, PD-1, and TNFRSF9. Based on an immunophenoscore to assess the response to immunotherapy developed by Charoentong et al. [26], we demonstrated that patients with CM with a low NRG score could benefit from anti-PD-1 or a combination of anti-CTLA-4 and anti-PD-1 immunotherapy. Furthermore, using an independent immunotherapeutic cohort (GSE91061) consisting of CM individuals receiving immune therapy, we again validated the predictive ability of NRG score to immunotherapeutic benefits of CM patients. The results indicated that individuals with low NRG scores showed a more prolonged survival compared with high score patients. Surprisingly, all individuals with clinical responses, including complete responses (CR)/partial responses (PR), belonged to the low NRG score group, which robustly validated that NRG score was an extremely sensitive predictor of immunotherapeutic benefits. Therefore, the NRG score could serve as an independent immunotherapeutic and prognostic indicator, thereby facilitating the identification of appropriate candidates for immunotherapy and the formulation of individualized therapeutic approaches.

Nevertheless, we should notice that these results are based on the TCGA and GEO public databases with a lack of biological validation. Our current CM cases are very limited and are far from sufficient to be used to conduct the clinical characteristic-related analysis. Furthermore, according to the above analysis, we believe that the relationships of necroptosis-related genes with immune features and underlying mechanisms of necroptotic stimulators promoting the antitumor immunity are indeed an interesting and promising research. We will pay more efforts on the clinical sample collection and follow-up and try to illustrate the underlying mechanisms in future studies.

## 5. Conclusion

Our present work revealed close correlations between expression patterns of necroptosis-related genes and tumor immune microenvironment. A scoring system, the NRG score, was established to comprehensively assess necroptosis-related gene expression patterns and the characterization of tumor immune microenvironment in individuals with CM, providing a basis for the determination of tumor immunophenotype and effective clinical practice. Furthermore, the NRG score has been shown to serve as a potential indicator to independently assess CM individuals' prognoses and to effectively estimate the response of individuals with CM to immunotherapy, all of which have been further validated, respectively, in our study based on other independent datasets. Finally, evaluating the expression patterns of necroptosis-related genes of individual tumors could contribute to enhancing our understanding of the comprehensive characteristics of tumor immune microenvironment and offer valuable insights for immunotherapy.

## Data Availability

The data used to support this study are included within the article.

## Supplementary Materials

Supplementary Materials Supplementary Figure 1. Consensus matrixes of screened genes. (A)–(D) Consensus matrixes of 67 necroptosis-related genes with the prognostic value for each *k*, showing the stability of clustering via 1000 iterations of hierarchical clustering (*k* = 2–5). (E)–(H) Consensus matrixes of necroptosis-related gene signatures for each *k*, showing the stability of clustering via 1000 iterations (*k* = 2–5). Supplementary Figure 2. Differences in expressions of necroptotic genes between mutants with normal samples. (A)–(Y) Difference in the necroptotic gene expression between the necroptotic gene wild and gene mutation groups. The interquartile range of data was indicated by the upper and lower ends of the boxes. The black dots in the boxes signified outliers, whereas the line in box means median value. “^*∗*^” represents obvious *P* value. Supplementary Figure 3. Distinct immune landscapes in the high and low RIPK3 expression groups. (A)–(E) Using various algorithms, including QUANTISEQ, CIBERSORT, CIBERSORT-ABS, EPIC, and MCPCOUNTER, validate the obvious positive relationship between the levels of RIPK3 with infiltrating levels of CD8+ T cells. (F)–(H) Differences in immune cell-infiltrating levels, immune-related pathways, expression level of antigen-presenting molecules, and immune checkpoints between the high and low RIPK3 expression groups. Supplementary Figure 4. Association of the NRG score with tumor mutation burden. (A), (B) Waterfall plot indicating that the TMB was constructed based on high and low NRG score patients. (C) Survival analysis of patients with low and high TMB. (D) Scatterplots demonstrated that the NRG score was uncorrelated with TMB. (E) The difference in TMB in the low and high NRG score groups.
