# Supplementary figures and images for: Expression Patterns of Necroptosis-Related Genes: Predicting Prognosis and Immunotherapeutic Effects in Cutaneous Melanoma
Source: J Oncol. 2022 Jul 14;2022:5722599. doi: 10.1155/2022/5722599 (PMC9303167; doi:10.1155/2022/5722599)

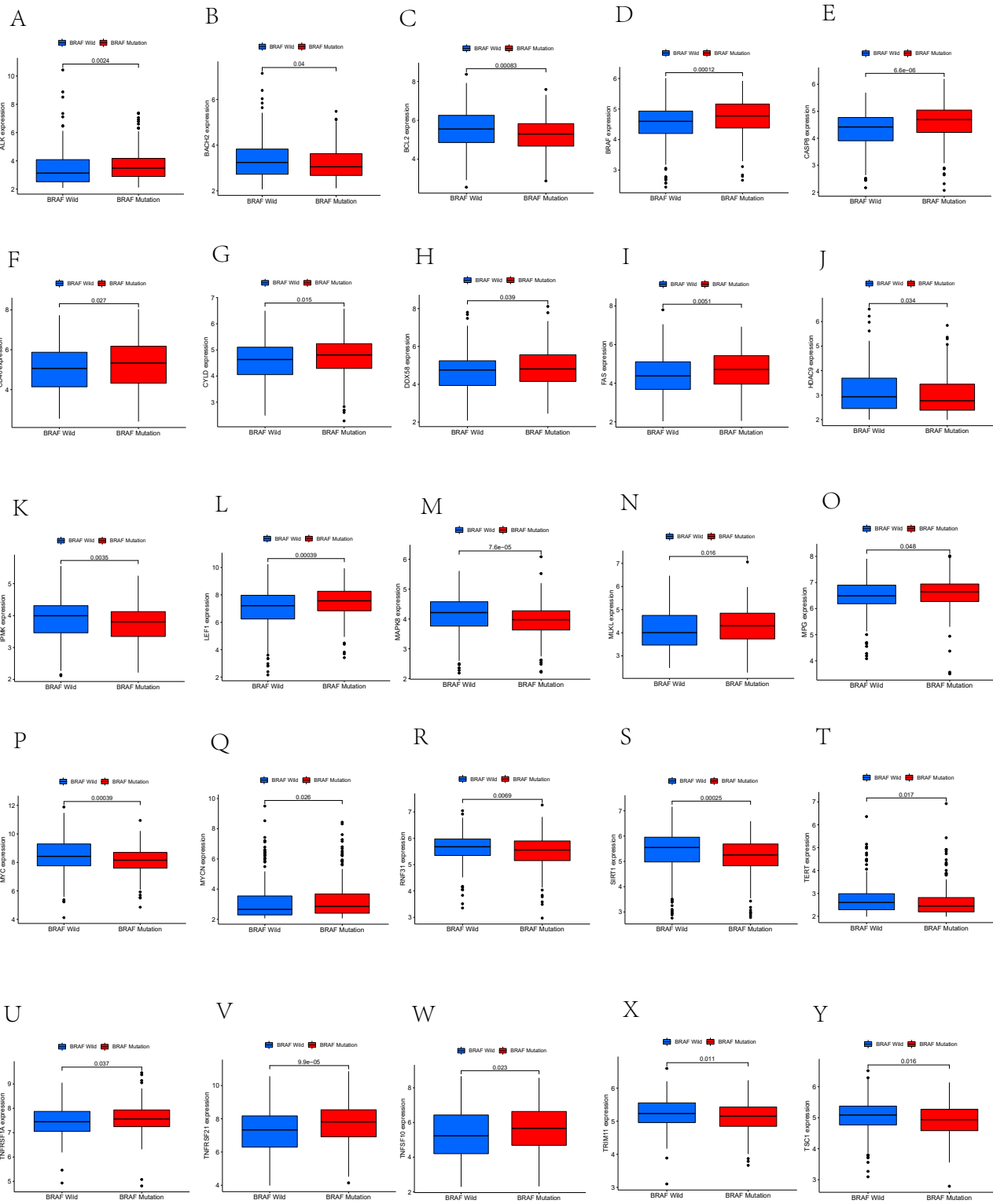

Supplement: Supplementary Materials — Supplementary Figure 1. Consensus matrixes of screened genes. (A)–(D) Consensus matrixes of 67 necroptosis-related genes with the prognostic value for each k, showing the stability of clustering via 1000 iterations of hierarchical clustering (k = 2–5). (E)–(H) Consensus matrixes of necroptosis-related gene signatures for each k, showing the stability of clustering via 1000 iterations (k = 2–5). Supplementary Figure 2. Differences in expressions of necroptotic genes between mutants with normal samples. (A)–(Y) Difference in the necroptotic gene expression between the necroptotic gene wild and gene mutation groups. The interquartile range of data was indicated by the upper and lower ends of the boxes. The black dots in the boxes signified outliers, whereas the line in box means median value. “∗” represents obvious P value. Supplementary Figure 3. Distinct immune landscapes in the high and low RIPK3 expression groups. (A)–(E) Using various algorithms, including QUANTISEQ, CIBERSORT, CIBERSORT-ABS, EPIC, and MCPCOUNTER, validate the obvious positive relationship between the levels of RIPK3 with infiltrating levels of CD8+ T cells. (F)–(H) Differences in immune cell-infiltrating levels, immune-related pathways, expression level of antigen-presenting molecules, and immune checkpoints between the high and low RIPK3 expression groups. Supplementary Figure 4. Association of the NRG score with tumor mutation burden. (A), (B) Waterfall plot indicating that the TMB was constructed based on high and low NRG score patients. (C) Survival analysis of patients with low and high TMB. (D) Scatterplots demonstrated that the NRG score was uncorrelated with TMB. (E) The difference in TMB in the low and high NRG score groups. [file 5722599.f1.zip › 5722599.f1/Supplement figure 2.pdf]

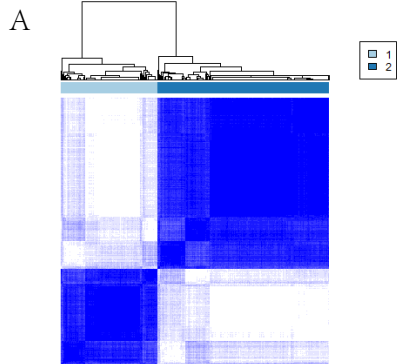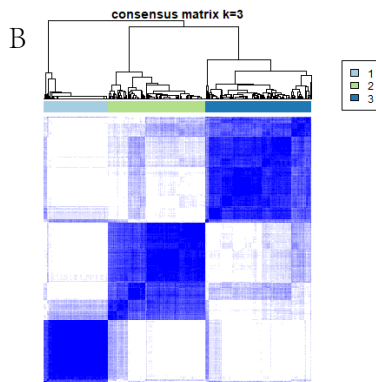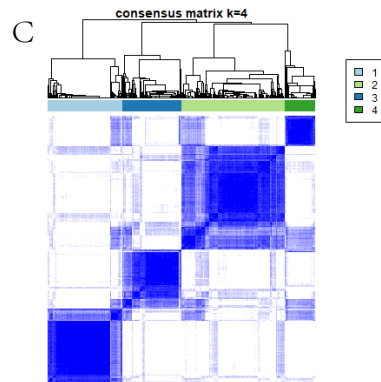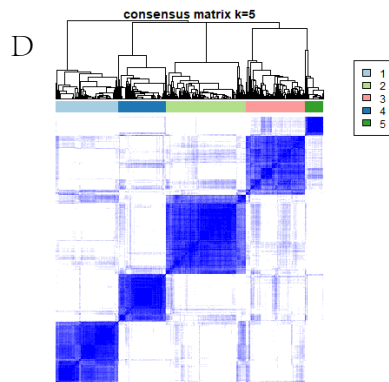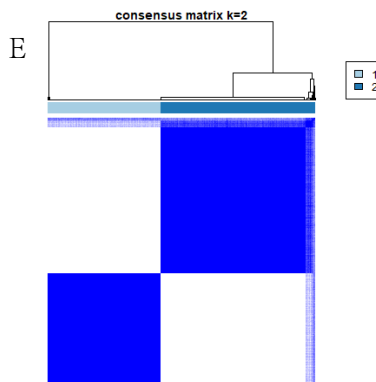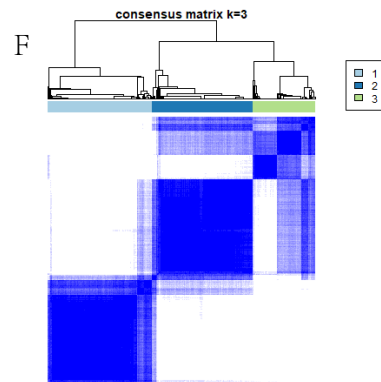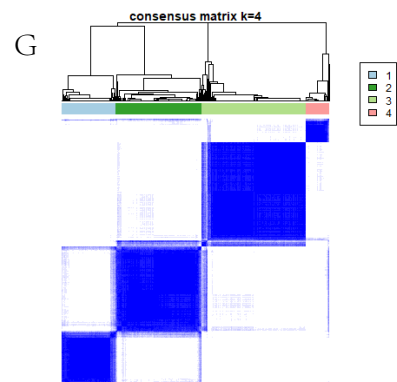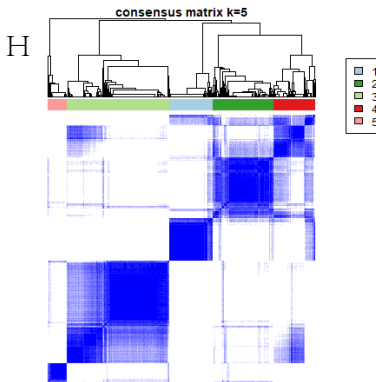

Supplement: Supplementary Materials — Supplementary Figure 1. Consensus matrixes of screened genes. (A)–(D) Consensus matrixes of 67 necroptosis-related genes with the prognostic value for each k, showing the stability of clustering via 1000 iterations of hierarchical clustering (k = 2–5). (E)–(H) Consensus matrixes of necroptosis-related gene signatures for each k, showing the stability of clustering via 1000 iterations (k = 2–5). Supplementary Figure 2. Differences in expressions of necroptotic genes between mutants with normal samples. (A)–(Y) Difference in the necroptotic gene expression between the necroptotic gene wild and gene mutation groups. The interquartile range of data was indicated by the upper and lower ends of the boxes. The black dots in the boxes signified outliers, whereas the line in box means median value. “∗” represents obvious P value. Supplementary Figure 3. Distinct immune landscapes in the high and low RIPK3 expression groups. (A)–(E) Using various algorithms, including QUANTISEQ, CIBERSORT, CIBERSORT-ABS, EPIC, and MCPCOUNTER, validate the obvious positive relationship between the levels of RIPK3 with infiltrating levels of CD8+ T cells. (F)–(H) Differences in immune cell-infiltrating levels, immune-related pathways, expression level of antigen-presenting molecules, and immune checkpoints between the high and low RIPK3 expression groups. Supplementary Figure 4. Association of the NRG score with tumor mutation burden. (A), (B) Waterfall plot indicating that the TMB was constructed based on high and low NRG score patients. (C) Survival analysis of patients with low and high TMB. (D) Scatterplots demonstrated that the NRG score was uncorrelated with TMB. (E) The difference in TMB in the low and high NRG score groups. [file 5722599.f1.zip › 5722599.f1/Supplementary figure 1 (1).pdf]

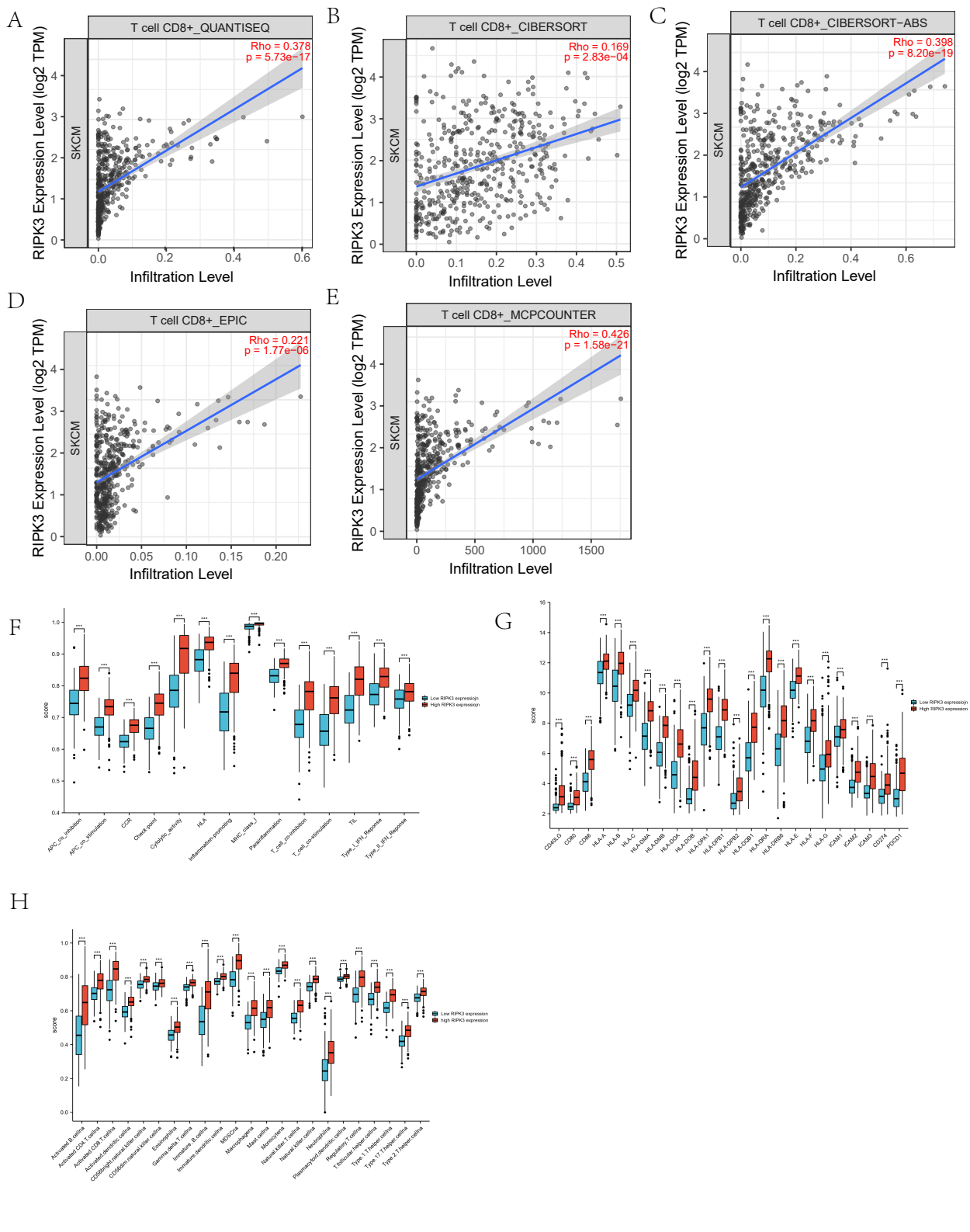

Supplement: Supplementary Materials — Supplementary Figure 1. Consensus matrixes of screened genes. (A)–(D) Consensus matrixes of 67 necroptosis-related genes with the prognostic value for each k, showing the stability of clustering via 1000 iterations of hierarchical clustering (k = 2–5). (E)–(H) Consensus matrixes of necroptosis-related gene signatures for each k, showing the stability of clustering via 1000 iterations (k = 2–5). Supplementary Figure 2. Differences in expressions of necroptotic genes between mutants with normal samples. (A)–(Y) Difference in the necroptotic gene expression between the necroptotic gene wild and gene mutation groups. The interquartile range of data was indicated by the upper and lower ends of the boxes. The black dots in the boxes signified outliers, whereas the line in box means median value. “∗” represents obvious P value. Supplementary Figure 3. Distinct immune landscapes in the high and low RIPK3 expression groups. (A)–(E) Using various algorithms, including QUANTISEQ, CIBERSORT, CIBERSORT-ABS, EPIC, and MCPCOUNTER, validate the obvious positive relationship between the levels of RIPK3 with infiltrating levels of CD8+ T cells. (F)–(H) Differences in immune cell-infiltrating levels, immune-related pathways, expression level of antigen-presenting molecules, and immune checkpoints between the high and low RIPK3 expression groups. Supplementary Figure 4. Association of the NRG score with tumor mutation burden. (A), (B) Waterfall plot indicating that the TMB was constructed based on high and low NRG score patients. (C) Survival analysis of patients with low and high TMB. (D) Scatterplots demonstrated that the NRG score was uncorrelated with TMB. (E) The difference in TMB in the low and high NRG score groups. [file 5722599.f1.zip › 5722599.f1/Supplementary figure 3 (1).pdf]

A

Altered in 281 (89.49%) of 314 samples.

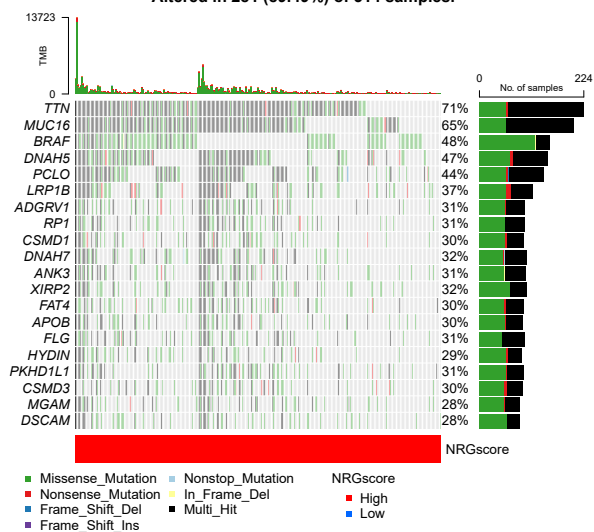

B

Altered in 133 (95%) of 140 samples.

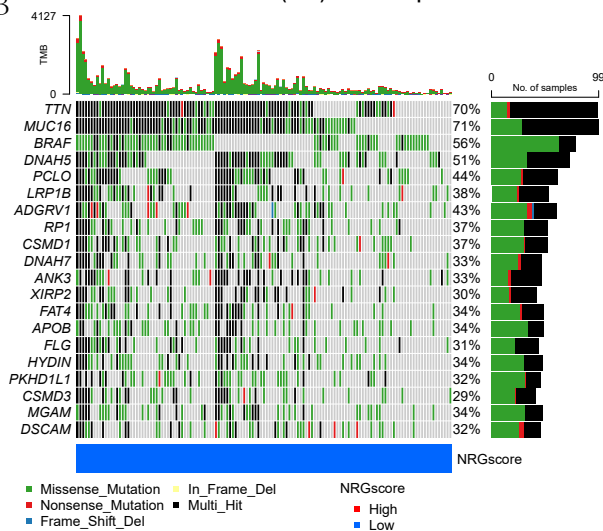

C

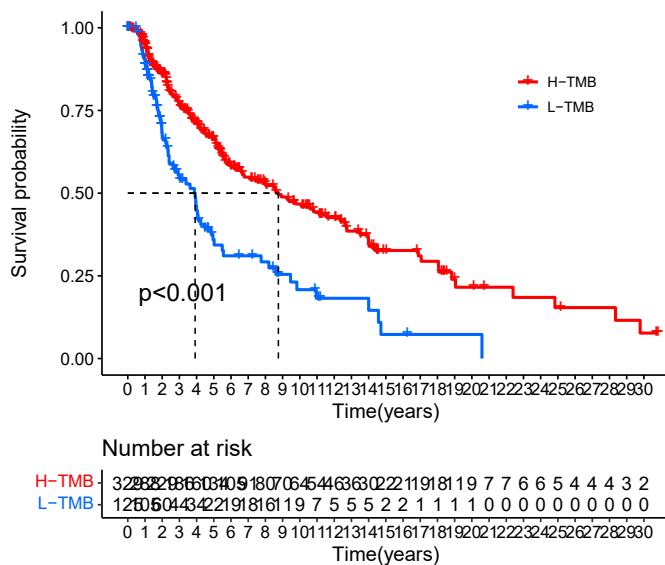

D

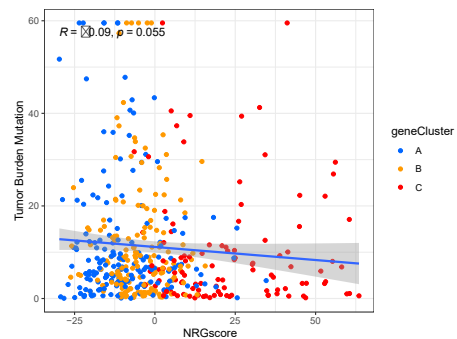

E

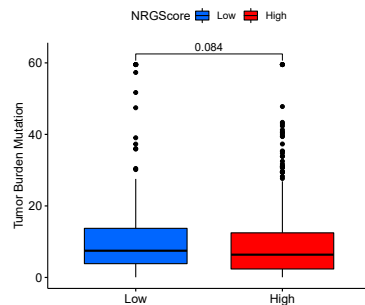

Supplement: Supplementary Materials — Supplementary Figure 1. Consensus matrixes of screened genes. (A)–(D) Consensus matrixes of 67 necroptosis-related genes with the prognostic value for each k, showing the stability of clustering via 1000 iterations of hierarchical clustering (k = 2–5). (E)–(H) Consensus matrixes of necroptosis-related gene signatures for each k, showing the stability of clustering via 1000 iterations (k = 2–5). Supplementary Figure 2. Differences in expressions of necroptotic genes between mutants with normal samples. (A)–(Y) Difference in the necroptotic gene expression between the necroptotic gene wild and gene mutation groups. The interquartile range of data was indicated by the upper and lower ends of the boxes. The black dots in the boxes signified outliers, whereas the line in box means median value. “∗” represents obvious P value. Supplementary Figure 3. Distinct immune landscapes in the high and low RIPK3 expression groups. (A)–(E) Using various algorithms, including QUANTISEQ, CIBERSORT, CIBERSORT-ABS, EPIC, and MCPCOUNTER, validate the obvious positive relationship between the levels of RIPK3 with infiltrating levels of CD8+ T cells. (F)–(H) Differences in immune cell-infiltrating levels, immune-related pathways, expression level of antigen-presenting molecules, and immune checkpoints between the high and low RIPK3 expression groups. Supplementary Figure 4. Association of the NRG score with tumor mutation burden. (A), (B) Waterfall plot indicating that the TMB was constructed based on high and low NRG score patients. (C) Survival analysis of patients with low and high TMB. (D) Scatterplots demonstrated that the NRG score was uncorrelated with TMB. (E) The difference in TMB in the low and high NRG score groups. [file 5722599.f1.zip › 5722599.f1/Supplementary figure 4.pdf]
